# Evaluation of 3D-Printed Microfluidic Structures for Use in AML-Specific Biomarker Detection of PML::RARA

**DOI:** 10.3390/ijms26020497

**Published:** 2025-01-09

**Authors:** Benedikt Emde, Karsten Niehaus, Lara Tickenbrock

**Affiliations:** 1 Department Hamm 1, Hamm-Lippstadt University of Applied Science, 59063 Hamm, Germany; benedikt.emde@hshl.de; 2 Proteome and Metabolome Research, Faculty of Biology, Bielefeld University, 33615 Bielefeld, Germany; kniehaus@cebitec.uni-bielefeld.de

**Keywords:** biomicrofluidics, personalized diagnostics and medicine, acute myeloid leukemia, additive manufacturing

## Abstract

An obstacle for many microfluidic developments is the fabrication of its structures, which is often complex, time-consuming, and expensive. Additive manufacturing can help to reduce these barriers. This study investigated whether the results of a microfluidic assay for the detection of the promyelocytic leukemia (PML)-retinoic acid receptor α (RARα) fusion protein (PML::RARA), and thus for the differential diagnosis of acute promyelocytic leukemia (APL), could be transferred from borosilicate glass microfluidic structures to additively manufactured fluidics. Digital light processing (DLP) and stereolithography (SLA) printers as well as different photopolymerizable methacrylate-based resins were tested for fabrication of the fluidics. To assess suitability, both print resolution and various physical properties, serializability, biocompatibility, and functionalization with biological molecules were analyzed. The results show that additively manufactured microfluidics are suitable for application in leukemia diagnostics. This was demonstrated by transferring the microfluidic sandwich enzyme-linked immunosorbent assay (ELISA) for PML::RARA onto the surface of magnetic microparticles from a glass structure to three-dimensional (3D)-printed parts. A comparison with conventional glass microstructures suggests lower sensitivity but highlights the potential of additive manufacturing for prototyping microfluidics. This may contribute to the wider use of microfluidics in biotechnological or medical applications.

## 1. Introduction

Microfluidics have rapidly revolutionized the diagnosis of diseases, genome sequencing, proteomics, and cell biology in recent years and also have a wide range of applications in other fields of biotechnology [1]. The success of microfluidics can be attributed to faster analysis times, a compact design, favorable manufacturing, operating costs, and mass applicability [2]. Based on that technology, micro-total-analysis-systems (µTAS) or lab-on-a-chip (LOC) systems are created by integrating several manual laboratory steps onto a microfluidic chip [3]. µTAS and LOC systems offer the possibility of the automated execution of complex biological assay protocols [3] through integration. Despite all these advantages, the potential of microfluidics is still not fully exploited [4] because the pathway from the research lab to the market is complex and expensive [5]. The gap between technical application and biological and biotechnological research must be reduced so that many promising approaches can find their way to the market [4]. Improvements in manufacturing methods, such as soft lithography, have also enabled rapid technical and academic developments, but the transfer to the production of commercial systems remains difficult [6].

Despite these obstacles, commercially available microfluidic systems as diagnostic tools are available on the market. The Claros1^®^ analyzer from OPKO Health, Inc. is a microfluidic-based rapid point-of-care test for the quantitative determination of PSA (prostatic-specific antigen) concentration in 10 µL blood samples [7]. Cytovale has developed IntelliSep, a microfluidic sepsis test that assesses the viscoelastic properties of leukocytes and can therefore evaluate the severity of sepsis [8]. Menarini Silicon Biosystems has launched DEPArray™, an image-based product that combines microelectronics and microfluidics for the isolation of circulating tumor cells (CTCs) [9,10].

Conventional manufacturing methods such as the production of microfluidics from polydimethylsiloxane (PDMS) using soft lithography require many manual process steps and special information structures and are therefore often expensive, not very user-friendly, and complex [5,11]. The additive manufacturing of microfluidic chips seems to be a promising approach, especially for the economic production of prototypes in one’s own laboratory [2,12]. While soft lithography is a multi-step 2D process, additive manufacturing methods can be used to produce 3D structures in a single step, while at the same time reducing production costs and time and increasing the flexibility of the fluidic designs [11]. In this study, we focus on the direct additive manufacturing of microfluidics, while indirect manufacturing using open-channel printing or tool-based manufacturing using molds is also possible [13].

High-resolution 3D printers represent a new technology for microstructure production, which is still largely unexplored for this application compared to existing technologies [2] but offers great potential for the rapid and automatable production of microfluidics [13,14] and can reduce the hurdles of production for research laboratories [13]. Additive manufacturing processes are characterized by the formation of structures through the layer-by-layer application of materials [15] and thus offer great design freedom in structuring. Automated manufacturing based on computer aided design (CAD) files gives life scientists easy access to microfluidic manufacturing. The rapid production and subsequent testing of fluidics enable faster development times through iterative design adaptation. This is also possible because there is no need for special infrastructure such as clean rooms [16]. Commercially available high-resolution 3D printers that cure photosensitive resin using light in the UV range are available for as little as a few thousand dollars. These printing techniques are called stereolithography (SLA) or digital light processing (DLP) and are often used for the production of microfluidics. In SLA printing, the light from the laser is projected via movable mirrors through a film of Teflon onto the photosensitive resin. [17]. This hardens at the exposed spot; then it is pulled out of the resin and the process is repeated. After printing, unpolymerized resin must be removed with 2-propanol. With DLP, the ultraviolet (UV) light is not projected by a laser but by a projector with a mirror array onto the surface to be illuminated [18]. DLP printers, therefore, have sharper edges and a higher speed due to the simultaneous projection of the entire mask. SLA and DLP printers have a higher resolution, smoother surface, and better sealing compared to fused deposition modeling (FDM) printers [2,16,19]. In the past, numerous studies were performed on direct the 3D printing of microfluidics with DLP printers. Macdonald et al. conducted a comparative study between FDM, DLP-SLA, and polyjet [20]. Shallan et al. used a DLP printer to produce 3D mixers, gradient, and droplet generators to detect nitrite in tap water, achieving print times of 12 min and unit costs of USD 1 [21]. Gong et al. were able to print 18 × 20 μm small microfluidic channels using a custom DLP 3D printer and an optimized resin of polyethylene glycol diacrylate-258 [22]. Hiniduma et al. have presented instructions for a 3D-printed microfluidic immunoarray for the Form3 SLA printer (Formlabs, Somerville, MA, USA) [23]. In a further study by Musgrove et al., SLA and DLP printers were compared for their suitability in the production of microfluidics using different resins [11]. Qiu et al. also demonstrated the use of DLP printers for the production of microfluidic chips for biomedical applications [24]. The rapid progress in additive manufacturing holds great future potential but also needs to be evaluated for various applications [2].

In our previous work [25], we developed a prototype for a microfluidic assay for the diagnosis of APL, a subtype of acute myeloid leukemia (AML). APL is characterized on a molecular level by the reciprocal translocation t(15;17) (q24;q21), which leads to the formation of the fusion protein PML::RARA [26]. Individuals with APL often exhibit severe coagulation disorders, posing a high risk of life-threatening bleeding in the brain as well as in the skin, mucous membranes, gastrointestinal tract, and lungs [27]. APL is considered a particularly aggressive and life-threatening form of AML [28]. The aim of APL therapy is curative. The therapy for APL consists of combination treatment with the vitamin A derivative all-trans retinoic acid (ATRA) and arsenic trioxide (ATO) [26,29]. With this chemotherapy-free combination treatment, remission rates of approx. 90% are achieved in APL today [30,31,32]. A fast diagnosis is crucial for initiating this targeted therapy, as APL has a high early mortality rate before or shortly after treatment initiation [33,34].

The detection of the t(15;17) (q24;q21) translocation is therefore considered diagnostically conclusive [35]. The transcript is detected on a nucleic acid basis using reverse transcriptase polymerase chain reaction (RT-PCR) or fluorescence in situ hybridization (FISH) [36]. RT-PCR has become the standard method for diagnosing APL, but it is very time-consuming and depends on the quality of the RNA in the patient sample. [36,37,38]. Our published assay detects the PML::RARA fusion protein at the protein level, not at the deoxyribonucleic acid (DNA) level. The PML::RARA fusion protein is detected using RARA and PML antibodies on the surface of magnetic particles by sandwich ELISA in less than 30 min [25]. The assay was validated both in a cell culture model and in primary patient samples, processed in microtiter format, and automated in a microfluidic chip [25].

The microfluidic structures used were produced out of borosilicate glass by photolithography and etching. That is very time-consuming and does not allow rapid design changes. Due to this circumstance, rapid changes in the fluidic structures were not possible during assay development. In the subsequent work, light-based 3D printers were evaluated for the production of microfluidic structures. The developed assay was transferred into the 3D-printed fluidics, and the results were compared with those from conventionally manufactured microfluidic structures made of glass. [25]

## 2. Results

**Figure 1:**
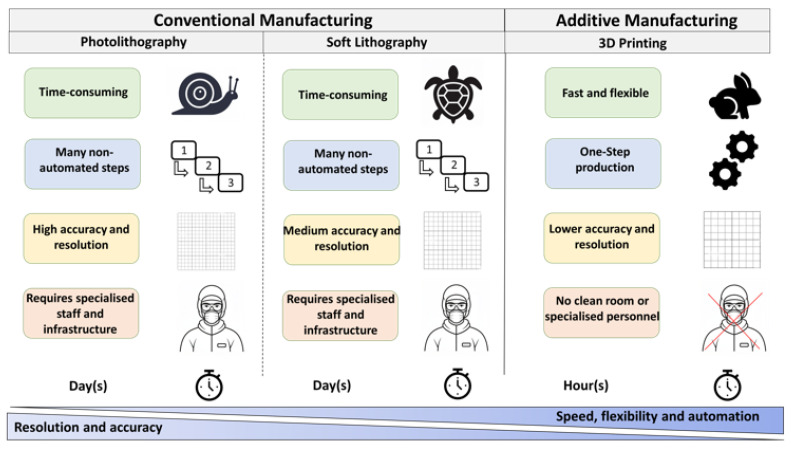
Comparison of conventional and additive manufacturing of microfluidics. Photolithography and soft lithography were selected as examples of conventional manufacturing; these are two commonly used processes. These processes are very time-consuming, consist of several non-automated steps, and require skilled staff and special infrastructure. The resolution and accuracy are highest with photolithography and decrease with 3D printing. Despite their lower resolution and accuracy, additive manufacturing methods have advantages in terms of production time and are much more flexible when structural changes are necessary. The number of manual process steps decreases from photolithography to soft lithography to additive manufacturing. The manufacturing process is automated and can be quickly adapted by anyone. Our own representation is based on [39].

In this work, the evaluation of 3D-printed microfluidic structures for biomicrofluidic assays in personalized diagnostics is presented, using acute promyelocytic leukemia as an example. Figure 1 compares the conventional manufacturing process with the additive process via 3D printing and illustrates the potential advantages and disadvantages of the two processes. While manufacturing with photo- or soft lithography has been well studied, accurate data for the fast-evolving additive manufacturing processes are often lacking. In general, it can be stated that photo- and soft lithography involve time-consuming, multistep [39], and manual processes with high accuracy and resolution [2]. On the other hand, additive manufacturing is fast and automatable, easily applicable even by inexperienced scientists, but exhibits deficiencies in resolution and accuracy [2].

**Figure 2:**
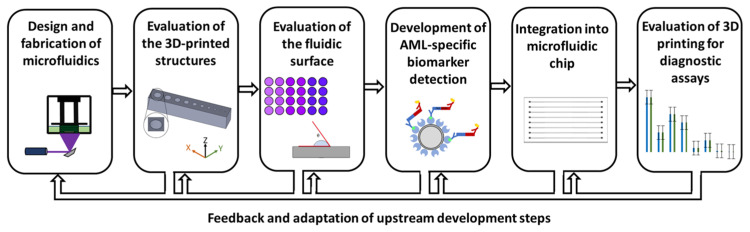
Evaluation of additive manufacturing for the production of microfluidics for diagnosis of acute promyelocytic leukemia by detection of PML::RARA. The process for the evaluation of 3D-printed microfluidics is divided into 6 steps. Specific work packages of the individual steps are described below. The arrows symbolize the progression of the process but also illustrate that each step can be adapted with the results of the following work packages. The first step is the structural design and production of the fluidics. A suitable 3D printing process, 3D printer, and materials must also be selected. Then, in step 2, the printing parameters and post-processing steps are defined and the printer resolution is evaluated. In step 3, the surface of 3D prints is characterized, sterilized, and tested for cell and biocompatibility. This is followed by the development of biomarker detection for PML::RARA. Samples are prepared and the detection assay is implemented and validated. In the fifth step, the biomarker detection is integrated into the 3D-printed microfluidic chip. Particular attention must be paid to the compatibility of the detection methodology. Step 6 concludes with an assessment of suitable 3D printers for manufacturing microfluidic chips by comparing the results from previous work [25].

To evaluate 3D-printed structures and their suitability as additive manufacturing for production, an initial set of requirements for the 3D-printed microfluidic structure was established, and the evaluation process was developed. First, 3D-printed fluidics must be able to produce channels with a diameter of ~500 µm, with consistent and repeatable quality. The surface should not induce background effects in diagnostic detection and should be compatible with all chemicals and biological molecules, being hydrophilic for optimal surface wetting. Additionally, the microfluidic structure should possess optical properties that allow for the detection of fluorescence intensity, enabling the detection of the specific biomarker for APL. Figure 2 visualizes the evaluation process, starting with the literature-based selection of 3D printing methods, printers, and materials.

For the printing of microfluidics, a resin-based 3D printing process was chosen. This method allows high-resolution and leak-free manufacturing, and it is cost-effective to acquire and operate [19,20]. For this purpose, both an SLA printer Form3b and a DLP printer MiiCraft Prime 110y, as well as various resins, were selected and compared with each other [11]. The resin-dependent printing parameters are shown in Table 1. The aim of the first setup was to determine the best printing process.

For the evaluation of X, Y, and Z resolutions, test bodies were printed and designed based on the model by Gensler et al. [19]. To assess the printability of channels, 2 mm thick test bodies with 10 channels ranging from 100 µm to 1000 µm in diameter were manufactured horizontally and vertically to the Teflon film. The channels were tested for printability, releasability, and leak-tightness. Initially, the SLA printer Form3b with a 405 nm laser was compared to the DLP printer MiiCraft Prime 110y with a 385 nm projector. The printing parameters from Table 1 were utilized. For evaluation, walls and trenches in the X, Y, and Z directions, as well as the diameters of the channels, were measured using a Keyence digital microscope at 100× magnification.

**Figure 3:**
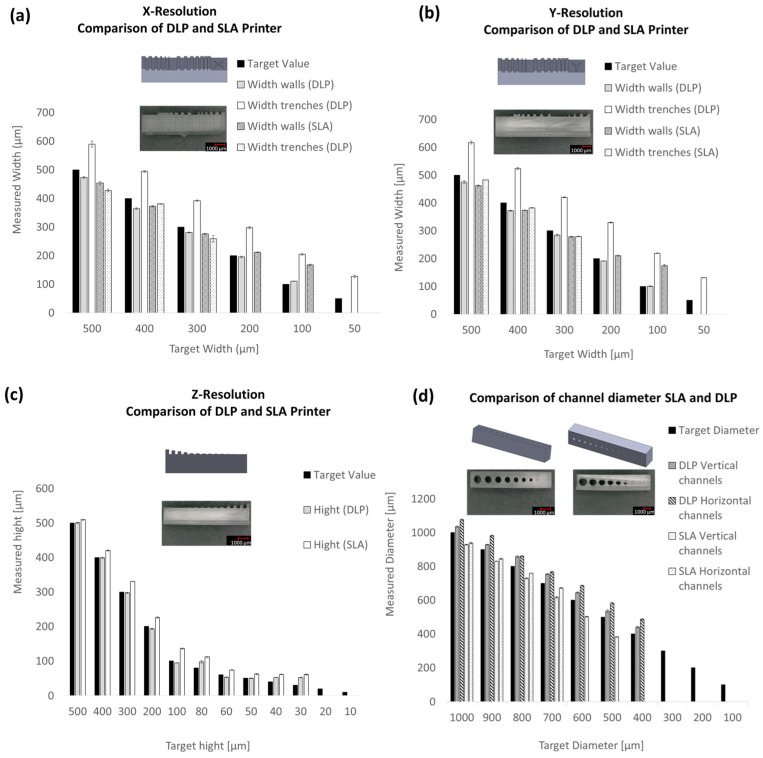
Comparison of X, Y, and Z resolutions, as well as the minimum achievable diameter, between Form3b (SLA printer) and MiiCraft Prime 110y (DLP printer). The results display the respective CAD model of the test object, microscopic images with 30-fold magnification of the printed structures, and measurements of the minimum widths and heights of walls. (**a**) Shows the results of comparing DLP and SLA printers in terms of X resolution, while (**b**) shows the Y resolution and (**c**) the Z resolution. Additionally, the minimum achievable diameter of channels with target diameters ranging from 100 to 1000 µm is presented (**d**). Three 3D prints were evaluated 3× microscopically (mean values).

The results in Figure 3, comparing the X, Y, and Z resolutions of the SLA and DLP printers, show that the DLP printer can print smaller structures in both the X and Y directions for both walls and trenches. With the Clear V4 resin from Formlabs, the DLP printer achieves approximately 100 µm width for walls and about 130 µm for trenches. In SLA printing, minimum widths of approximately 175 µm for walls and even only 280 µm for trenches are achieved. In Z resolution, the DLP system achieves minimum heights of about 50 µm, while the SLA system can print structures with a minimum height of about 60 µm. Form3b also faces limitations in the printability of channels. Horizontal channels with a diameter of approximately 670 µm and vertical channels with a diameter of approximately 380 µm can be printed. The minimum target diameter was set at 500 µm. In comparison, the MiiCraft Prime 110y is able to print target diameters of 400 µm, which have horizontal diameters of approximately 480 µm and vertical diameters of around 440 µm. Based on these results, the DLP method was chosen for further experiments.

**Figure 4:**
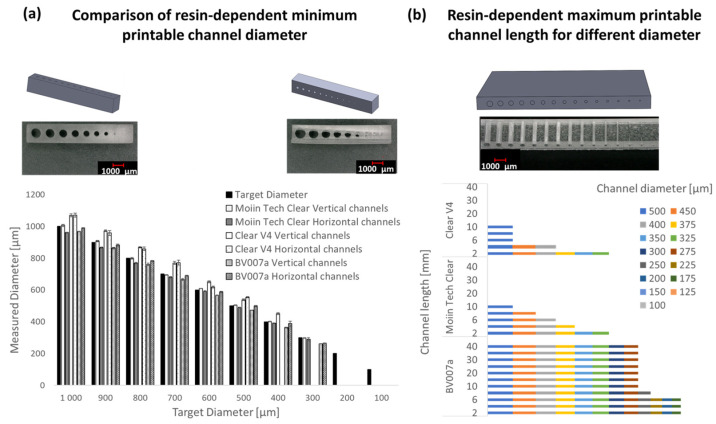
Comparison of the minimum printable channel diameters with the MiiCraft Prime 110y for the resins Clear V4, BV007a, and Tech Clear (**a)**. The results indicate that the smallest diameters can be achieved with BV007a at approximately 260 µm, followed by Tech Clear at around 295 µm, and Clear V4 at approximately 440 µm. (**b**) shows the results of the evaluation of the maximum channel length depending on the used diameter. With the BV007a resin, 40 mm long channels with a minimum target diameter of 275 µm can be produced, while Clear V4 and Moiin Tech Clear enable maximum 10 mm long channels with a diameter of 325 µm.

In addition to the 3D printing technique, the choice of resin plays a significant role in terms of the minimum achievable resolution and the appearance of prints. Because the SLA printer Form3b by Formlabs has a closed material system, two additional resins, BV007a and Moiin Tech Clear, were compared with Clear V4 resin on the DLP printer MiiCraft Prime 110y for microstructure printing. For this comparison, a test body with 10 channels ranging from 100 to 1000 µm in diameter was used. The results are presented in Figure 4, indicating that both horizontal and vertical channels of approximately 295 µm can be printed with Tech Clear resin, while with BV007a, channels of around 260 µm are achievable. In contrast, the Clear V4 resin only allows channels with a minimum of approximately 440 µm (a). Subsequently, for the resins Clear V4, Tech Clear, and BV007a, the maximum channel length was determined in relation to the channel diameter using the MiiCraft Prime 110y (b). Finally, 40–2 mm long channels with diameters of 500–100 µm were printed and then inspected under a digital microscope for their continuity.

In general, the results show that longer channels can be printed with larger diameters. With the Clear V4 resin, a maximum of 10 mm long channels can be printed at a diameter of 500 µm; 2 mm long channels are still 325 µm in diameter. The results with Moiin Tech Clear are identical for the maximum channel length of 10 mm at 500 µm diameter and 2 mm at 325 µm diameter; 6 and 8 mm long channels are still continuous up to 450 and 400 µm in diameter, whereas these are no longer continuous with Clear V4 resin. The best aspect ratio of canal diameter and canal length is achieved with the BV007a resin; 40 mm long canals can be achieved with diameters of 500–275 µm. Subsequently, a drop-off can be observed from a diameter of 250 µm, and only 8 mm long continuous channels can be produced; 2 mm long channels can be produced with a minimum target diameter of 175 µm. BV007a was chosen for further experiments.

**Figure 5:**
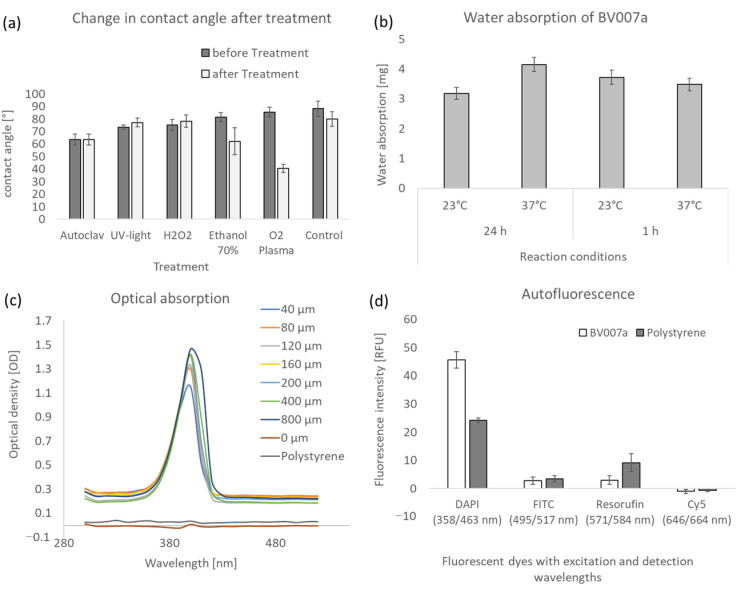
Physical and optical properties of Resin BV007a (**a**) Shows the contact angles of the BV007a resin before and after sterilization using an autoclave, UV light, hydrogen peroxide, 70% ethanol, and oxygen plasma. Untreated BV007a served as a control. The average contact angle of untreated BV007a is approx. 77°. A significant change in the contact angle only occurs when treated with oxygen plasma to a contact angle of approx. 40°. The surface becomes more hydrophilic. Contact angle measurements were carried out on three workpieces each, and the mean value was calculated. The water absorption (**b**) was determined for BV007a for 24 h and 1 week at room temperature and 37 °C. The maximum water absorption is 4 mg, which is a maximum of 2%. The optical absorbance of BV007a (**c**) was determined as a function of the layer thickness (40–800 µm) for the wavelengths 300–500 nm by means of an absorption scan. Polystyrene was used as a control. The optical density is plotted against the wavelength and decreases with lower film thickness. The optical density reaches its highest value between 385 and 405 nm, depending on the layer thickness between 1 and 1.4 OD. The autofluorescence of BV007a (**d**) was determined as an example for excitation and detection wavelengths of dyes DAPI, FITC, Resorufin, and Cy5 and is at a maximum of ~45 RFU for DAPI (358/463 nm). BV007a shows no autofluorescence for the tested wavelengths. The contact angle, water absorption and autofluorescence were measured on three independent samples in triplicates. The error bars show the standard deviation of the measurements.

To assess the suitability of the 3D-printed microfluidics made from BV007a for compatibility with biological assays, important physical and optical properties were initially evaluated. The wettability of the surface often plays a crucial role in microfluidics, as it influences the flow behavior of biological materials. The surface was characterized by measuring the contact angle (Figure 5a). The results show that contact angles range between 65° and 88°, indicating a hydrophilic and wettable surface. Water absorption is an important property in the field of microfluidics, as high water absorption can affect the concentration of reagents in microchannels. The results for water absorption are presented in Figure 5b, showing that water absorption for 24 h and one week at temperatures of 23 °C and 37 °C is at a maximum of 4 mg, corresponding to a percentage of maximum ~1.75%. The transmission of BV007a was determined according to method 4.4, depending on the material thickness, in the wavelength range of 300–800 nm. The results are shown in Figure 5c for the wavelength range of 300–500 nm, covering the peak with an optical density (OD) of maximum 1.46 OD at a wavelength of 400 nm. The optical density steadily increases from 350 nm and reaches a maximum of 0.25 OD at 430 nm before decreasing again. Compared to polystyrene, BV007a exhibits an approximately 10-times-higher optical density and the absorption peak at 400 nm is not present in polystyrene. The OD of BV007a also increases with increasing layer thickness. The results indicate that absorption measurements are possible with BV007a but may be overlaid by a background of about 0.2 OD, which can affect the sensitivity of the measurements. In evaluating the applicability of BV007a-printed fluidics for fluorescence-based assays, the structures underwent autofluorescence measurements using dyes such as DAPI, FITC, Resorufin, and Cy5. BV007a demonstrated the highest recorded value of approximately 40 relative fluorescence units (RFU), compared to polystyrene’s measurement of around 25 RFU at the excitation and emission wavelengths of DAPI. Importantly, BV007a exhibited no autofluorescence across all tested wavelength combinations, thereby avoiding any potential interference with fluorescence-based assays.

**Figure 6:**
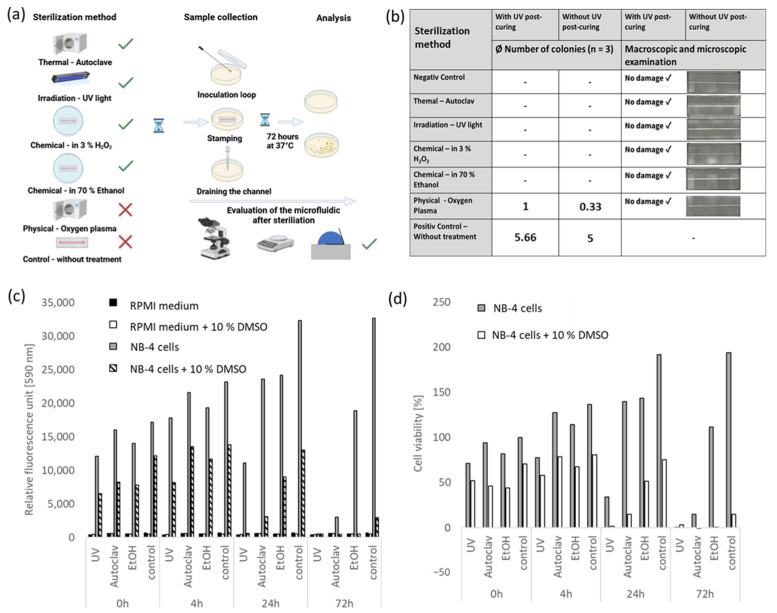
Evaluation of the sterilizability and biocompatibility of 3D prints using BV007a. Sterilization methods used are autoclaving, UV light, chemical hydrogen peroxide, and ethanol as well as oxygen plasma (**a**). The sterilized samples were placed on LB agar dishes, drained, and swabbed with an inoculation loop. The LB agar dishes were subsequently incubated for 72 h at 37 °C. The results show that all methods except oxygen plasma are suitable for the sterilization of BV007a (**b**). No colonies grew from the dishes. Optical inspection of the component showed that sterilization did not cause any damage (**b**). The bio- and cell compatibility of the material BV007a was evaluated using the alamarBlue™ HS assay. For this purpose, discs were printed, sterilized, and placed in a 96-well plate. Subsequently, 1 × 10^5^ NB-4 cells in 100 µL were added to each well and incubated for 0, 4, 24, and 72 h in an incubator. After the incubation time, 10% alamarBlue™ HS reagent was added and incubated again at 37 °C for 2 h. The fluorescence intensity was determined at 590 nm. The results show that BV007a has no acute cell toxicity (**c**). The values of four-hour incubation are consistently higher than after direct viability measurement. Over 24 h, the intensity values for the autoclave and ethanol sterilization methods continue to increase slightly, while UV light is significantly lower. After 72 h, hardly any living cells could be detected during sterilization using UV light and autoclave, while a cell viability of over 100% could be measured with sterilization with ethanol and the control (**d**).

In addition, the sterilizability, as well as bio- and cell compatibility of 3D prints made from BV007a, was also tested. The methodology is described in Section 4.3 and Section 4.5. Figure 6a illustrates the schematic process of sterilization using various methods. Subsequently, the sterility test was conducted through swabs on lysogeny broth (LB) agar, a visual inspection was conducted for integrity, and the weight and contact angle were checked.

In addition, the sterilizability, as well as bio- and cell compatibility of 3D prints made from BV007a, was also tested. The methodology is described in Section 4.3 and Section 4.5. Figure 6a illustrates the schematic process of sterilization using various methods. Subsequently, the sterility test was conducted through swabs on lysogeny broth (LB) agar, a visual inspection was conducted for integrity, and the weight and contact angle were checked.

Figure 6a outlines the schematic process of sterilization and evaluation of 3D-printed test bodies. The results of sterilization in Figure 6b show that successful sterilization was achieved through autoclaving, UV light irradiation, 70% ethanol, and 3% hydrogen peroxide. No colonies of microorganisms grew on the LB agar plates. Oxygen plasma, however, exhibited no sterilizing effect but rather a cleaning effect, as approximately one colony less than the control (about five colonies) could be detected. None of the sterilization methods resulted in visual observable damage or weight changes in the prints. The contact angle only changed significantly during treatment in oxygen plasma, decreasing from approximately 85° to about 40°.

Subsequently, the sterilized prints were tested for cell and biocompatibility using the alamarBlue™ HS reagent. The results (Figure 6c,d) show that BV007a has no short-term toxicity on the leukemia NB-4 cells. Comparing fluorescence intensity values at 0 and 4 h of incubation with BV007a, it is observed that intensity increases by at least 35% regardless of the sterilization method or control, indicating an increase in cell count. From 4 to 24 h, fluorescence intensity slightly increases in autoclaved and UV-treated samples, while it decreases slightly with UV light irradiation. For the cell viability (%) (Figure 6d), the value (NB-4 polystyrene 0 h) is assumed to be 100%. Based on this value, the fluorescence values are converted into percentages. Negative values are assumed to be 0% cell viability. The largest increase is observed in the control on polystyrene. After 72 h, the cell viability stagnates at approximately 232% in the control, while there are no significant differences between sterilization methods. Ethanol shows a slight decrease (24 to 36 h) from 172% to about 104%, while autoclaved samples experience a significant drop from about 167% to about 9.6% RFU. UV-treated samples show no higher fluorescence intensity than controls without NB-4 cells or with NB-4 cells and 10% DMSO. NB-4 cells with 10% DMSO exhibit significantly lower RFU values at 0 h compared to NB-4 cells without DMSO. The results indicate that the sterilization method has an impact on the biocompatibility of BV007a. Ethanol sterilization shows biocompatibility after both 24 and 72 h, while autoclaved BV007a has little effect on cells up to 24 h. UV-light sterilized BV007a only shows a short-term positive development in RFU values after 4 h.

**Figure 7:**
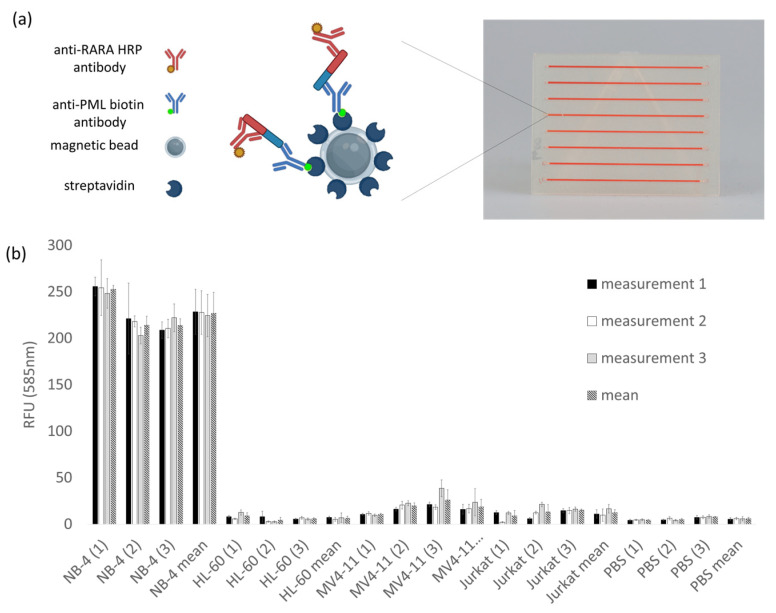
Magnetic bead-based sandwich ELISA for PML::RARA in a 3D-printed microfluidic chip. The figure schematically shows the detection of the fusion protein and the additively manufactured microfluidic chip made of BV007a (**a**). The results show the fluorescence intensities of the sandwich ELISA on magnetic particles in the 3D-printed microfluidic chip made of BV007a. Lysates of the cell cultures NB-4, HL-60, MV4-11, and Jurkat with a total protein concentration of 100 µg/mL were used for the analysis. PBS served as a protein-free control. From each cell line, three different cell lysates, labelled in the figure with the numbers 1, 2 and 3 in rectangular brackets, were measured in triplicate (**b**). The bars represent the standard deviations of the triplicate measurements, which are highest for NB-4 with a mean of ~227 RFU. HL-60 shows a mean value of ~6.5 RFU, MV4-11 of ~19 RFU, Jurkat with 12.4, and PBS with 5.8 RFU.

After evaluating the biocompatibility of resin BV007a, we wanted to transfer the microfluidic detection of PML::RARA for the diagnosis of acute promyelocytic leukemia to 3D-printed microfluidic chips made of BV007a. The results of the previous experiments are considered in this experimental design. The measurement of the fluorescent dye resorufin at 585 nm detection wavelength was established on the Tecan reader and adapted to the 3D-printed chip. Equal concentrations of the fluorescent dye are measured in all eight channels with similar RFU values (unpublished data) to validate the measurement method. The results of the bead-based sandwich ELISA in the 3D-printed microfluidic system were performed on the cell culture model. Figure 7 schematically illustrates the detection of the fusion protein PML::RARA on the surface of the magnetic beads as well as the 3D-printed microfluidic chip (a) in which the assay was performed, and the results of the sandwich ELISA (b) are shown. Cell lysates of the cell lines NB-4 (PML::RARA positive), HL-60, MV4-11, and Jurkat were used with a concentration of 100 µg/mL. phosphate-buffered saline (PBS) served as a control without proteins. From each cell line, three different cell lysates were measured in triplicate. The results show mean values of triplicate measurements from three different lysates of each cell culture. PML::RARA-positive NB-4 lysates show a mean value of ~227 RFU, while the negative cell lysates show significantly lower values with approx. 6.5 RFU for HL-60, ~19 RFU for MV4-11, and ~12 RFU for Jurkat. PBS shows values of approx. 5.8 RFU. The test for significance between the individual samples was carried out using Tukey’s test. This demonstrates that the NB-4 values significantly differ from the values of samples lacking PML::RARA, such as HL-60, MV411, Jurkat, and PBS.

## 3. Discussion

In the current work, additive manufacturing was evaluated as a possibility for the production of microfluidics. As an example, the microfluidic detection of the fusion protein PML::RARA for the diagnosis of APL in the glass microfluidic chip is transferred to microfluidic chips from the 3D printer. It is important to consider the strengths and weaknesses of the process, along with its possibilities and limitations. In a highly technological field such as biomicrosystems technology, it is often not possible for one technology to offer advantages in all applications. A differentiated discussion forms the basis for the evaluation.

The evaluation process, which is important for comparability with other studies, is shown in Figure 2. The focus here was on resin-based 3D printers. The choice of printing process and 3D printer was made between the SLA printer Form 3B and the DLP printer MiiCraft Prime 110y. The resolution in the X, Y, and Z directions was evaluated by printing walls and trenches as well as channels with different diameters with the resin Clear V4. The production of walls and trenches plays a particularly important role in the indirect production of microfluidics. In this process, a mold is additively manufactured for molding with PDMS, and the structure is then closed via bonding [13]. The printing of channels is particularly relevant for the direct production of microchannels [9]. The results showed that the DLP printer MiiCraft Prime can produce smaller structures with the same resin compared to the SLA Printer Form3B and that the deviation of the actual dimension from the target dimension is also smaller. A detailed view of the minimum printable channel diameters shows that the DLP printer can produce the 400 µm target diameter channels both vertically and horizontally oriented to the build platform, while the SLA printer can only print horizontal channels up to 700 µm target diameter and vertical channels up to 500 µm target diameter. This may be due in particular to the free parameterization capability of the DLP printer. While only prefabricated print protocols can be used in the Form3B, an optimized result can be achieved with the MiiCraft Prime 110y by adjusting the exposure intensity, time, and print speed used depending on the layer thickness.

In general, the alignment of the channels in the printing area is also an important aspect. While channels aligned vertically to the printing bed and printing platform achieve a smaller diameter and a smaller deviation from the target diameter, horizontal alignment of the channels is still recommended. Other factors such as adhesion to the baseplate, the printing time, and the functionality of the prints also play a decisive role in the alignment process. Adhesion to the printing platform is highest when the largest surface of the component adheres to the build platform. A microfluidic chip that contains 40 mm long channels, and therefore dimensions of 50 mm × 35 mm × 2 mm, requires approx. 25-times-longer printing time with the vertical alignment of the channels, which is made up of the required layers. While a 50 mm long component consists of 1250 layers with a height of 40 mm, a 2 mm component requires 50 layers with a height of 40 mm. Each layer increases both the exposure time and the mechanical stress on the component [11], which leads to a rougher surface structure. It therefore makes sense to choose an alignment with as few layers as possible in order to optimize the printing process [11]. Suitable compensation methods must be developed for small channels to achieve better printing accuracy [24]. The inlets and outlets of the channels are positioned orthogonally to the channels so that uncured resin can flow off during the printing process.

A literature-based preselection was made for the resin to print microfluidics [6] and then evaluated according to the aspects of the minimum printable channel diameter and the maximum printable channel length depending on the diameter. The resin Clear V4, Moiin Tech Clear, and BV007a were printed with the DLP printer MiiCraft Prime 110y. The smallest channel diameters of ~260 µm can be achieved with the BV007a resin, while 295 µm can be achieved with Moiin Tech Clear and 440 µm with only Clear V4. The different diameters can be explained by the composition of the resin. After printing, uncured resin is flushed out of the microchannels with 2-porpanol and compressed air. In resin-based 3D printing, the removal of trapped uncured resin from the channels determines the minimum achievable channel diameter. Shallan et al. found that channels smaller than 250 μm in diameter were clogged with uncured resin [21].

Printing with BV007a resin also enables the longest clearable channels with the smallest measurable diameter. This is a decisive parameter for printing microfluidic structures. The fact that BV007a shows significantly better results here may be related to the low viscosity of 75–100 mPa·s. Clear V4 and Moiin Tech Clear have viscosities around ten times higher at 850–900 mPa·s and 800 mPa·s, respectively. From the results of the maximum printable channel length from Figure 4, it can be deduced that BV007a is best suited to produce microfluidic channels with a length of 40 mm. In the following, BV007a is further discussed for its suitability for the production of microfluidics.

The physical and optical properties play a decisive role in microfluidic systems and therefore the evaluation of these is crucial for an assessment. The influence of subsequent processing steps (cleaning, coating, and sterilization) on the material properties must also be considered. The contact angle for measuring wettability was measured after and before sterilization. The values show that a significant change is only caused by oxygen plasma. With an average contact angle of ~77°, the surface of structures made of BV007a can be classified as hydrophilic. In contrast, hydrophobization is described in the literature through the use of fluorinated oil (FC-40) [40]. The water absorption of BV007a is also very low with less than 2%, which underlines its suitability for use in microfluidics. Increased water absorption would influence the assay liquids in terms of their substance concentration or viscosity, which in turn would have an effect on the desired reaction.

The optical properties of microfluidic chips are particularly important for combination with optical detection systems. PDMS is often combined with very good optical properties. Polystyrene is also frequently used in forms of multiwell plates for absorption and fluorescence intensity measurements. Autofluorescence is often a critical point with polymers. The fluorescence intensity of BV007a is low for the excitation and emission wavelengths of the dyes DAPI, FITC, Resorufin, and Cy5 and comparable to that of polystyrene (Figure 5). Comparable autofluorescence to PDMS is also described in the literature [11]. Autofluorescence should be specifically considered when selecting a resin assay.

The transparency of a microfluidic is crucial to be able to observe the microfluidic channels under a microscope or to use absorption-based detection methods. In terms of optical density, BV007a shows values about 10 times higher than polystyrene and an absorption peak between 365 and 415 nm, where the OD values increase depending on the layer thickness to 1.14 OD (40 µm layer) and 1.46 OD (800 µm layer). The layer thickness seems to have an influence on the absorption maximum in the peak at ~400 nm, but outside this peak, the OD of very thin BV007a layers of 40 µm is about 10 times as much as polystyrene. The absorption is due to the photo absorber of BV007a. If the resin did not absorb light in this wavelength range, its suitability as a photo resin would be inconceivable. For very sensitive measurements, the use of BV007a as a structural material is not recommended. Post-processing steps such as grinding, polishing, and coating can slightly improve the optical density. As the production of the fluids should be kept as simple as possible, these methods were not used here. However, BV007a does not achieve values comparable to those of polystyrene and glass by reducing the layer thickness [11].

Structures made of BV007a can be sterilized using an autoclave, UV light, ethanol, and 3% hydrogen peroxide. All methods do not result in any optically visible damage, changes in weight, or changes in contact angle. Sterilization using oxygen plasma is not suitable. Sterilizability was tested on a fluidic chip with a channel 0.5 mm in diameter and 40 mm long. The channel was rinsed with PBS after sterilization and then incubated on an LB agar plate. Whether each sterilization also achieves more complex fluidic systems cannot be evaluated conclusively. These procedures would have to be re-evaluated for more complex structures. The biocompatibility test with BV007a resin was carried out with PML::RARA-positive cell line NB-4. In combination with sample preparation by cell lysis and the detection of the fusion protein PML::RARA, the leukemic blasts come into contact with the microfluidic chip. Since this assay can be performed within a few hours, short-term cell compatibility plays a particularly important role; 10% DMSO was used as a positive control. Cell viability is significantly lower for all controls with 10% DMSO than without. In general, the difference in cell viability between NB-4 cells and NB-4 cells + 10% DMSO increases over the duration from 0 to 72 h, which shows the cytotoxic effect. Cell viability was also examined in dependence on sterilization methods. The results suggest that sterilization techniques have an effect on the biocompatibility of BV007a (Figure 6d). Especially sterilization by UV light shows (after 24 h with about 75%) lower cell viability, while BV007a sterilized with autoclave and ethanol shows 167% and 172%, which is much higher cell viability. After 72 h, only BV007a sterilized with ethanol showed cell viability values over 100% (104%). This could mean that sterilization with ethanol causes cytotoxic substances to be sponged out of the BV007a resin. For usage in cell-based biomicrofluidic assays that require higher cell and biocompatibility, further experiments should be carried out with regard to sponging. For our application, sterilization with 70% ethanol for 30 minutes is recommended due to the cell viability results discussed here.

In additive-manufactured microfluidics with BV007a, the detection of PML::RARA by sandwich ELISA via magnetic particles was chosen as a biological assay. The presence of fusion protein PML::RARA is one major point of diagnostics in APL. Translocation (15;17) is present in approximately 95% of APL patients. The sandwich method increases the specificity of the detection. A specific PML antibody (PG-M3) binds at the PML region of the fusion protein and an RARα antibody (C1) at the RARA region. The PML antibody is conjugated with biotin, which binds with streptavidin to magnetic particles. The RARA antibody is conjugated with HRP for enzymatic detection. For the successful detection of the fusion protein, both antibodies have to bind. NB-4 cell lysate is used as a positive sample, as the presence of the translocation t(15;17) was detected by RT-PCR [41]. HL-60 and MV4-11 are also AML cell lines, but they do not express PML::RARA fusion protein. Jurkat, an ALL cell line, is used as a further negative control. The results show that the additively manufactured microfluidic channels with a diameter of 500 µm and a length of 40 mm were suitable for carrying out this assay. Many microfluidic systems have dimensions in the range of 100 µm or smaller. Structure sizes of less than 100 µm could not be produced from any of the resins used with the DLP printer MiiCraft Prime 110y. For biomicrofluidic assays that require structure sizes below 100 µm, production using other methods such as custom 3D printers or individual resin formulation would have to be evaluated. Some groups have already additively manufactured microfluidics at these scales [22,42,43]. In this study, channels with a length of 40 mm and a minimum diameter of 275 µm could be produced. Similar dimensions were also achieved in other studies using commercially available 3D printers and resins [11,24].

**Figure 8:**
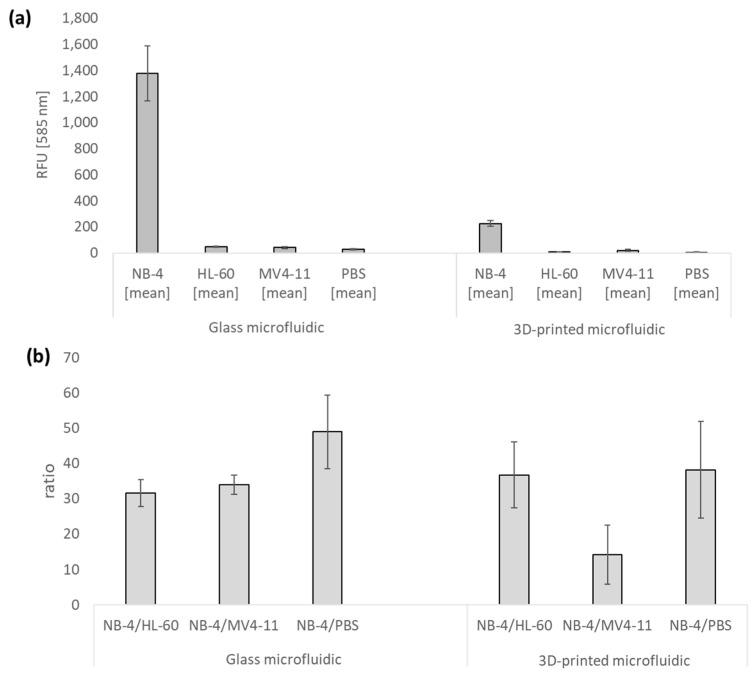
Comparison between glass and 3D-printed microfluidic chips for the detection of PML::RARA. The figure shows the comparison between the results of the detection of PML::RARA in a glass and 3D-printed microfluidic chip (BV007a). (**a**) shows RFU values for NB-4, HL-60 MV4-11 and PBS for glass and 3D-printed microfluidics. (**b**) shows calculated ratio between positive control (NB-4) and negative controls HL-60, MV4-11 and PBS. The error bars show the standard deviation of three independent measurements in triplicates. The ratio indicates the factor of the RFU values between the positive control and the different negative controls. Data for the detection of glass microfluidic chips are taken from Emde et al. [25].

The results of the previously published assay show that the assay can be transferred from glass microfluidics to additively manufactured structures. NB-4 shows mean RFU values of ~220 whereas the negative controls show at least about 14-times-lower values. It is noticeable that RFU values of NB-4 in glass with ~1400 RFU are about 10 times higher than in 3D-printed fluidics with ~220 RFU. Since a total protein concentration of 100 µg/mL was used in both tests, the concentration can be excluded as the cause of this difference. Considering the optical properties of BV007a, an inhibition of the signal intensity is expected, which seems to be responsible for the lower RFU values in 3D-printed structures. Based on these results, it is assumed that the detection limit of PML::RARA using bead-based ELISA in 3D-printed fluidics is lower than in glass microfluidic chips. Considering the ratios (Figure 8b), it is noticeable that these are roughly comparable with NB-4/HL-60 and NB-4/PBS glass and BV007a fluidics at 30–50 times. The highest ratio of ~49 is achieved with NB-4/PBS in glass microfluidics. All ratios are at a minimum of ~14, which indicates a specific detection of PML::RARA of the assay. The comparison of the ratios for NB-4/PBS buffer in conventionally manufactured structures made of glass (approx. 49-fold) and additively manufactured structures (38-fold) indicates that the non-specific binding of assay components can also be effectively prevented on the modified surface of the additively manufactured microfluidic chips so that these do not cause false-positive signals.

We used additive manufacturing technology to produce a microfluidic chip with eight channels for the detection of PML::RARA in less than ~12 min for a few dollars. Microfluidic chips can be manufactured in a very short time, while often necessary structural adjustments can be implemented in just a few minutes. The additive manufacturing of microfluidics can make an important contribution to new developments in diagnostic tests, and, therefore, it plays a crucial role in the future of medicine.

## 4. Materials and Methods

PML antibody (PG-M3) (Santa Cruz Biotechnology, Dallas, TX, USA), RARA antibody (C-1) (Santa Cruz Biotechnology, Dallas, TX, USA), Roti-MagBeads Streptavidin (Carl Roth, Karlsruhe, Germany), Low-Cross-Buffer (CANDOR Bioscience, Wangen im Allgäu, Germany), bovine serum albumin (BSA) (Carl Roth, Karlsruhe, Germany), Roti quant universal (Carl Roth, Karlsruhe, Germany), Biotin-NHS (Thermo Fisher, Waltham, MA, USA), PBS-Buffer (AppliChem, Darmstadt, Germany), Tween 20 (Bio-Rad, Hercules, CA, USA), NB-4 cell line (Leibniz Institute DSMZ-German Collection of Microorganisms and Cell Cultures GmbH, Braunschweig, Germany), HL-60 cell line (Leibniz Institute DSMZ-German Collection of Microorganisms and Cell Cultures GmbH, Braunschweig, Germany), MV4-11 cell line (Leibniz Institute DSMZ-German Collection of Microorganisms and Cell Cultures GmbH, Braunschweig, Germany), Jurkat cell line (Leibniz Institute DSMZ-German Collection of Microorganisms and Cell Cultures GmbH, Braunschweig, Germany), IP-Lysis-Buffer (Thermo Fisher, Waltham, MA, USA), protease inhibitor cocktail (Thermo Fisher, Waltham, MA, USA), FBS (Gibco Thermo Fisher, Waltham, MA, USA), Pen/Strep (Gibco Thermo Fisher, Waltham, MA, USA), Roswell Park Memorial Institute (RPMI) Medium (Gibco Thermo Fisher, Waltham, MA, USA), QuantaRed™ Enhanced Chemifluorescent HRP Substrate (Thermo Fisher, Waltham, MA, USA), syringe pumps (World Precision Instruments, Sarasota County, FL, USA), syringes (B. Braun Melsungen, Melsungen, Germany), 3-way valve (IDEX Health & Science, Oak Harbor, WA, USA), motor selection valve (IDEX Health & Science, Oak Harbor, WA, USA), tubes (Carl Roth, Karlsruhe, Germany), connectors (Carl Roth, Karlsruhe, Germany), multiwell plate reader infinite M1000Pro (Tecan, Männedorf, Switzerland), microscope axiovert 40 CFL (Carl Zeiss, Oberkochen, Germany), multiwell plates (Thermo Fisher, Waltham, MA, USA), Tecan i-control 1.9.17.0 (Tecan, Männedorf, Switzerland), LabVIEW 2018 (National Instruments, Austin, TX, USA), Microsoft Office (Microsoft, Redmond, WA, USA), magnet neodym 40 × 10 × 5 mm (Webcraft GmbH, Gottmadingen, Germany), pipettes (1–10 µL, 10–100 µL, and 100–1000 µL) (Eppendorf, Hamburg, Germany), Heraeus Megafuge universal centrifuge ((Thermo Fisher, Waltham, MA, USA), BCA assay (Thermo Fisher, Waltham, MA, USA), MiiCraft Prime 110y 385 nm (MiiCraft, Hsinchu City, Taiwan), Form3b (FormLabs Inc., Somerville, MA, USA), Form Cure (FormLabs Inc., Somerville, MA, USA), Form Wash (FormLabs Inc., Somerville, MA, USA), Resin BV007a (MiiCraft, Hsinchu City, Taiwan), Moiin Tech Clear (MOIIN Resins, Hamburg, Germany), Clear V4 Resin (FormLabs Inc., Somerville, MA, USA), Digital Microscope VHX-5000 (Keyence, Osaka, Japan), HERAsafe sterile workbench (Thermo Fisher, Waltham, MA, USA), low-pressure plasma system (Diener Plasma GmbH & Co. KG, Ebhausen, Germany), 70% ethanol (Carl Roth, Karlsruhe, Germany) analyzing scale (KERN & SOHN GmbH, Balingen, Germany), UV exposure chamber (Purion GmbH, Zella-Mehlis, Germany), alamarBlue™ HS (Thermo Fisher, Waltham, MA, USA), DMSO (AppliChem, Darmstadt, Germany), LB-Medium (Carl Roth, Karlsruhe, Germany), Petri dish (Carl Roth, Karlsruhe, Germany), 2-propanol (Carl Roth, Karlsruhe, Germany), picker (MiiCraft, Hsinchu City, Taiwan), resin tank (MiiCraft, Hsinchu City, Taiwan), PreForm (FormLabs Inc., Somerville, MA, USA), utility (MiiCraft, Hsinchu City, Taiwan), SolidWorks (Dassault Systèmes, Vélizy-Villacoublay, France), contact angle-measuring device OCA 15 (DataPhysics Instruments GmbH, Filderstadt, Germany), autoclave (Systec GmbH & Co. KG, Linden, Germany), hydrogen peroxide 3% (Carl Roth, Karlsruhe, Germany), inoculation loop (Carl Roth, Karlsruhe, Germany), and ultrasonic bath (EMAG AG, Mörfelden-Walldorf, Germany) were acquired.

### 4.1. Three-Dimensional Printing of Test Bodies and Microfluidic Components

All 3D-printed parts were designed with SolidWorks, converted into a standard transformation language (STL) file, and then processed in the printer-specific slicer software, utility (MiiCraft, Hsinchu City, Taiwan) and PreForm (FormLabs Inc., Somerville, MA, USA) with the print parameters and divided into layers. The print parameters depend on the printer and resin used. The print parameters for the MiiCraft Prime 110y are listed in Table 1. The standard FormLabs parameters were used for Form3b from FormLabs.

After printing, the 3D-printed parts were cleaned in Form Wash for 15 min in 2-propanol then removed from the printing platform and cleaned again in 2-propanol in an ultrasonic bath three times for 5 min between the washing cycles with compressed air. Finally, the dry prints were post-cured in Form Cure at room temperature for 5 min per side.

### 4.2. Evaluation of the Minimum Printable Feature Size

To analyze the resolution, minimum printable feature size, and shape, the test bodies were documented after printing using the digital microscope VHX-5000 (Keyence, Osaka, Japan) and measured with the VHX-5000 Software (version number 1.3.2.4) (Keyence, Osaka, Japan). Firstly, the DLP printer MiiCraft Prime was compared with the SLA printer Form3b. As Form3b can only process FormLabs resin, the Clear Resin V4 from (FormLabs Inc., Somerville, MA, USA) is processed on both printers. The most suitable resin is then selected by processing Clear V4 (FormLabs), Clear Tech (MOIIN Resins, Hamburg, Germany), and BV007a (MiiCraft, Hsinchu City, Taiwan) on the open DLP printer material. The test structure is shown in Figure 3 (Section 2). The X, Y, and Z resolutions were printed with a test body with trenches and walls in the 3 room directions in the build space. The minimum printable channel diameter was determined with a 2 mm thick part with channel diameters of 1000–100 µm in steps of 100 µm. The component was manufactured with vertical and horizontal channels. The maximum printable channel length was evaluated as a function of the diameter. The evaluated diameters are 500, 450, and 400 µm from 375 to 100 µm; 25 µm steps were used. The channel lengths were 2, 4, 6, 8, 10, 15, 20, 25, 30, 35, and 40 mm. The patency was determined on the digital microscope VHX-5000. All experiments were carried out in triplicate.

### 4.3. Evaluation of Sterilizability

A test body measuring 20 × 7 × 2 (L × W × H) mm with a 0.5 mm diameter channel was developed for sterilization evaluation. Test bodies were produced using the DLP printer MiiCraft Prime 110y (MiiCraft, Hsinchu City, Taiwan) and the material BV007a (MiiCraft, Hsinchu City, Taiwan). Sterilization was evaluated using five methods. These are autoclave, UV light irradiation, 70% ethanol, 3% hydrogen peroxide, and oxygen plasma. The components were sterilized for 15 minutes at 121 °C in steam, sterilized for ~1 minute at a dose of 600 J/m^2^ in the UV sterilization chamber, immersed in 70% ethanol or 3% hydrogen peroxide for 15 minutes, and then rinsed with PBS and dried. Sterilization in oxygen plasma was carried out for 2 minutes per side with 40 kHz, 60 W, and 6.5 sccm oxygen flow at 1 ± 0.17 mbar process pressure. Three test bodies were used for each type of sterilization. After sterilization, test bodies were examined microscopically for damage, the weight was measured before and after sterilization, and the contact angle was determined. An untreated test specimen was used as a positive control. Packed Petri dishes served as a control. To check sterility, the test specimens were pressed onto an LB agar plate after treatment, wiped with an inoculation loop, the channel was rinsed with PBS through a cannula, and 2 drops were dripped onto the LB agar plate. The LB agar plates were incubated at 37 °C for 72 h. The colonies on the LB agar plates were counted for analysis. All experiments were carried out in triplicate.

### 4.4. Evaluation of Physical Properties

The 3D-printed test specimens were characterized in terms of wettability, water absorption, optical properties transmission, and autofluorescence. The wettability was determined by analyzing the surface contact angle using the contact angle-measuring device OCA series (DataPhysics Instruments GmbH, Filderstadt, Germany).

Water absorption of printed BV007a was determined based on the DIN EN ISO 20795-1 [44]. For this purpose, 15 × 1 mm cylinders were printed and dried to a constant weight at 40 °C (m_1_). The samples were then incubated in 10 mL of water at room temperature and 37 °C. The samples were then blotted dry and dried for 15 s and weighed again (m_2_). The water uptake was calculated according to Equation (1), the equation for the calculation of the water adsorption:

(1) $$WS=\frac{m_{2}-m_{1}}{V}\;$$

Biomicrofluidics are often used in combination with optical detection methods such as microscopic or spectroscopic methods. Transparency is important for the optical inspection of microfluidic chips as well as for microscopic or absorption-based detection in the system. The compatibility of the optical detection methods with the 3D-printed fluidics made of methacrylate-based resins must first be tested. For this purpose, both the autofluorescence and the transmission were determined as a function of the layer thickness of BV007a. For measurements, a sample holder was developed for the multiwell plate reader infinite M1000Pro (Tecan, Männedorf, Switzerland), which has the basic dimensions of a 96-well plate and a sample holder. Samples can be inserted into this for measurement. The sample is a strip with measuring points the size of a cavity of a 96-well plate with a diameter of 6.4 mm. The layer thickness varies in individual wells. Different samples were printed for different post-processing methods. Absorption scans to determine transmissions were carried out in a wavelength range of 300–800 nm. The autofluorescence was determined for the excitation and emission wavelengths of frequently used fluorescent dyes, which cover a wavelength range from 358 to 664 nm. Excitation and emission wavelengths and associated fluorescent dyes are listed in Table 2.

### 4.5. Evaluation of Cell and Biocompatibility

The cell and biocompatibility of BV007a were assessed through direct cell contact with the APL cell line NB-4, depending on the sterilization method. The biocompatibility test followed the guidelines of DIN EN ISO 10993-5 [45], which focuses on “tests for in vitro cytotoxicity”. To conduct this evaluation, 6 mm diameter discs were 3D printed, sterilized, and then placed in standard polystyrene microtiter plates using vacuum aspiration. A working volume of 100 µL was employed, and 1 × 10^6^ NB-4 cells were pipetted into the wells of the BV007a plates. Controls included RPMI medium, RPMI medium + 10% DMSO, 1 × 10^6^ NB-4 cells + 10% DMSO, and a 96-well polystyrene microwell plate with and without BV007a discs. Incubation periods for the samples were set at 0, 4, 24, and 72 h at 37 °C and 5% CO2. Following the incubation, 10 µL of the cell viability reagent alamarBlue™ HS was added, and the mixture was incubated for 2 h at 37 °C and 5% CO_2_. Subsequently, the fluorescence intensity of the resulting reaction product, resazurin, was measured at 560/590 nm. The percentage cell viability was calculated by dividing the fluorescence intensity values of the test groups by the control group (0h, polystyrene) and then multiplying by 100.

### 4.6. Sample Selection and Preparation

Different leukemic cell lines were used on cell culture samples. Cell line NB-4 with the translocation t(15;17) validated by RT-PCR was used as a positive control, while AML cell lines HL-60 and MV4-11 together with ALL cell line Jurkat formed negative controls. The cell lines HL-60 (ACC 3), MV4-11 (ACC 102), NB-4 (ACC 207), and Jurkat (ACC 282) were obtained from Leibniz Institute DSMZ-German Collection of Microorganisms and Cell Cultures GmbH, Braunschweig, Germany. The cell lines are listed under the specified Accession Numbers (ACC Numbers) in the database CellDive. Cells were cultured under optimized conditions in RPMI medium with 10% FCS and 1% penicillin/streptomycin. For cell lysis, Thermo Fisher’s IP Lysis Buffer was used. The protein concentration was determined by the BCA assay and stored at −80 °C until further analysis.

### 4.7. APL-Specific Biomarker Detection in 3D-Printed Microfluidic Structures

As an APL-specific biomarker, the PML::RARA fusion protein was analyzed using a sandwich ELISA on the surface of magnetic beads. The magnetic particles are coated with streptavidin, and the biotinylated anti-PML antibody binds to the streptavidin surface of the magnetic particles and with its epitope to the PML part of the fusion protein PML::RARA. The anti-RARA antibody is conjugated to horseradish peroxidase (HRP) and binds specifically to the RARA part of the fusion protein. The substrate conversion of QuantaRed™ catalyzed by HRP generates a fluorescent signal in the presence of PML::RARA. Cell lysates were used with a concentration of 100 µg/mL. In addition, 3D-printed microfluidic structures were fabricated with the MiiCraft Prime 110y in BV007a and also consist of eight 40 mm long channels. These have a diameter of 500 µm. Inlets have an internal diameter of 1 mm. Detection in a 3D-printed microfluidic chip was performed in a multifunctional microplate reader. A special layout was designed for the measurement at 20 different positions in one microfluidic channel. The values of the 20 measured values were determined for the evaluation.

## 5. Conclusions

In the present work, the transfer of the bead-based microfluidic detection of PML::RARA for the diagnosis of APL from glass microfluidics to 3D-printed structures was investigated. This evaluation can be understood as a proof of concept to transfer further biomicrofluidic assays from conventionally manufactured structures to 3D-printed structures in the future. For this purpose, the DLP process was initially selected and previously compared with the SLA process using the Form3b and MiiCraft Prime 110y printers as examples. BV007a proved to be the most suitable resin, as this resin could be used to produce the channels with the smallest diameter and the greatest length. Moreover, 3D-printed BV007a resin can be classified as hydrophilic due to its surface properties and its optical properties for fluorescence spectroscopic detection. In addition, the BV007a resin exhibits good sterilizability with various methods and shows short-term compatibility with leukemic cells, which is generally sufficient for diagnostic systems in the field of leukemia. The DLP 3D printer MiiCraft Prime could be used to produce microfluidic structures for the detection of the fusion protein PML::RARA for the diagnosis of acute promyelocytic leukemia quickly and cost-effectively. The production of a microfluidic chip takes about 12 min and costs about USD 1.50. Other groups also report the fast and cost-effective production of microfluidic chips using DLP 3D printers [21]. Compared to the results from microfluidic chips made of glass, DLP printing is a suitable method for the production of transparent microfluidic structures. The additive manufacturing of microfluidics offers particular advantages in the rapid prototyping of lab-on-a-chip systems for point of care (POC) diagnostics of diseases. The additive manufacturing of microfluidics makes it possible to produce, evaluate, and redesign prototypes and small series in just a few hours or days. The digital CAD or STL files can also be made available to researchers at other research institutions for further development or testing. The automated production process also requires few financial material and personnel resources and can be adapted quickly.

## Figures and Tables

**Table 1:** Resin-specific printing parameters for MiiCraft Prime 110y 385.

Resin	Layer Height (µm)	Cure Time (s)	Peel Speed	Gap Adj.(mm)	Base Layer	Base Cure Time (s)	Buffer Layer	Light Engine Power (%)
Clear V4 (FormLabs)	50	3.50	Slow	0.00	1	7.00	4	58
Moiin Tech Clear (Moiin)	50	4.25	Slow	0.00	1	35.00	5	110
BV007a (MiiCraft)	50	2.20	Fast	0.00	1	25.00	2	92

**Table 2:** Excitation and emission wavelengths for autofluorescence measurements.

Associated Fluorescent Dye	Excitation Wavelength	Detection Wavelength
DAPI (4′,6-Diamidin-2-phenylindol)	358 nm	463 nm
FITC (Fluorescein isothiocyanate)	495 nm	517 nm
Resorufin	571 nm	584 nm
Cy5	646 nm	664 nm

## Data Availability

The dataset will be made available upon request from the authors. The raw data supporting the conclusions of this article will be made available by the authors upon request.
